# Creatine salts provide neuroprotection even after partial impairment of the creatine transporter

**DOI:** 10.1016/j.neuroscience.2016.02.038

**Published:** 2017-01-06

**Authors:** E. Adriano, P. Garbati, A. Salis, G. Damonte, E. Millo, M. Balestrino

**Affiliations:** a Department of Neuroscience, Ophthalmology, Genetics, Maternal-Infantile Sciences, University of Genova, Largo Paolo Daneo 3, 16132 Genova, Italy; b Department of Hearth Environmental and Life Science (DISTAV), University of Genova, Corso Europa 26, 16132 Genova, Italy; c Department of Experimental Medicine, Section of Biochemistry, University of Genova, Viale Benedetto XV 1, 16132 Genova, Italy; d Center of Excellence for Biomedical Research, University of Genova, Viale Benedetto XV 5, 16132 Genova, Italy

**Keywords:** BBB, blood-brain barrier, CrT, creatine transporter, GPA, guanidine acetic acid, TLC, thin layer chromatography, creatine, creatine gluconate, creatine ascorbate, creatine glucose, neuroprotection, creatine transporter deficiency

## Abstract

Creatine, a compound that is critical for energy metabolism of nervous cells, crosses the blood-brain barrier (BBB) and the neuronal plasma membrane with difficulty, and only using its specific transporter. In the hereditary condition where the creatine transporter is defective (creatine transporter deficiency) there is no creatine in the brain, and administration of creatine is useless lacking the transporter. The disease is severe and incurable. Creatine-derived molecules that could cross BBB and plasma membrane independently of the transporter might be useful to cure this condition. Moreover, such molecules could be useful also in stroke and other brain ischemic conditions. In this paper, we investigated three creatine salts, creatine ascorbate, creatine gluconate and creatine glucose. Of these, creatine glucose was ineffective after transporter block with guanidine acetic acid (GPA) administration. Creatine ascorbate was not superior to creatine in increasing tissue creatine and phosphocreatine content after transporter impairment, however even after such impairment it delayed synaptic failure during anoxia. Finally, creatine gluconate was superior to creatine in increasing tissue content of creatine after transporter block and slowed down PS disappearance during anoxia, an effect that creatine did not have. These findings suggest that coupling creatine to molecules having a specific transporter may be a useful strategy in creatine transporter deficiency. In particular, creatine ascorbate has effects comparable to those of creatine in normal conditions, while being superior to it under conditions of missing or impaired creatine transporter.

## Highlights

• Creatine is a compound that is critical for energy metabolism of nervous cells.
• Creatine absence due to deficit of creatine transporter causes severe brain symptoms.
• Creatine crosses BBB and neuronal membrane slowly, and only using its transporter.
• Creatine derivatives may cross BBB and neuronal membrane without the transporter.
• Creatine derivatives may be a useful strategy in creatine transporter deficiency.

## Introduction

Creatine or Methyl Guanidino-Acetic Acid is an amino acid that is central to the energetic metabolism of the cells, particularly those with high energy requirements like neurons (Béard and Braissant, 2010). Inside the cells creatine is reversibly phosphorylated to phosphocreatine, with which it is in constant equilibrium. The functions of this creatine/phosphocreatine system are twofold. Under physiological conditions it moves ATP from the site where it is synthesized (the mitochondrion) to the cytoplasmic sites where it is utilized (mainly the plasma Na/K-ATPase). Under pathological conditions of energy deprivation, it provides rapidly available energy to replenish failing energy reserves. The body, even the brain, can synthesize creatine but is also takes it up with the diet. To reach the brain, creatine synthesized by the body or taken up with the diet must cross the blood-brain barrier (BBB), and to do so it needs a specific transporter (creatine transporter or CrT) codified by the gene SLC6A8 (Christie, 2007). Once across the BBB, it needs again the same transporter to cross the cell plasma membrane and enter brain neurons (Lunardi et al., 2006). Exogenous creatine may be useful for human therapy in two main disease groups:

1. Cerebral ischemia and stroke (Balestrino et al., 2002, Prass et al., 2006, Perasso et al., 2013).
2. Hereditary diseases with primary deficiency of brain creatine (Stockler, 1997). Among them, creatine can be used to restore its brain content in those conditions where its synthesis is impaired (GAMT or AGAT deficiency), but it is not useful in the hereditary creatine transporter deficiency (OMIM 300352) where cerebral synthesis of creatine is not sufficient to provide normal brain content. In the latter case, lacking the transporter it cannot be taken up by the BBB nor by cerebral cells. Thus, creatine transporter deficiency is currently incurable (Nasrallah et al., 2010).

A possible way of administering creatine in creatine transporter deficiency could be to modify the creatine molecule in such a way as to create a molecular structure that can cross the BBB and at the same time maintain a biological activity similar to that of creatine (Balestrino et al., 2002). While such molecules have indeed been researched, even by our laboratory (Perasso et al., 2008, Perasso et al., 2009, Adriano et al., 2011, Enrico et al., 2013), no fully satisfactory treatment is available. For this reason, we shifted our attention to the formation of creatine salts formed by two moieties, one of which is a molecule that has its own transporter. In this way, the salt may be transported across both BBB and cell plasma membrane by the latter transporter, that is unimpaired by creatine transporter deficiency. Once in the cells, the salts should be split and free creatine should remain. These compounds may therefore be useful for the treatment of creatine transporter deficiency. Furthermore, they could also be used to treat cerebral ischemia and stroke (Perasso et al., 2013). Specifically, we tested three different creatine derivatives: (1) a salt formed from creatine and ascorbate (a molecule that has been before suggested as a vector across the BBB – Dalpiaz et al., 2005); (2) a salt formed from creatine and glucose; and (3) a salt from creatine and D-gluconic acid (this latter salt, commercially available, should use the glucose transporter to cross the BBB).

As a model, we used mouse hippocampal slices. Although no BBB obviously exists in this *in vitro* model, creatine still needs its transporter to cross the neuronal plasma membrane and enter cells, thus compounds’ efficacy under conditions of creatine transporter impairment may be tested in this model (Lunardi et al., 2006). As a gauge of efficacy, we tested the ability of creatine and of creatine-derived compounds to:

1. Delay the disappearance of evoked electrical potentials during anoxia, a well-known effect of creatine that we used before in our research (Perasso et al., 2008) and
2. Increase the tissue concentrations of creatine and phosphocreatine.

We carried out the above investigations both under control conditions and after pharmacological block of the creatine transporter.

## Results

### Creatine-derived compounds

**Fig. 1:**
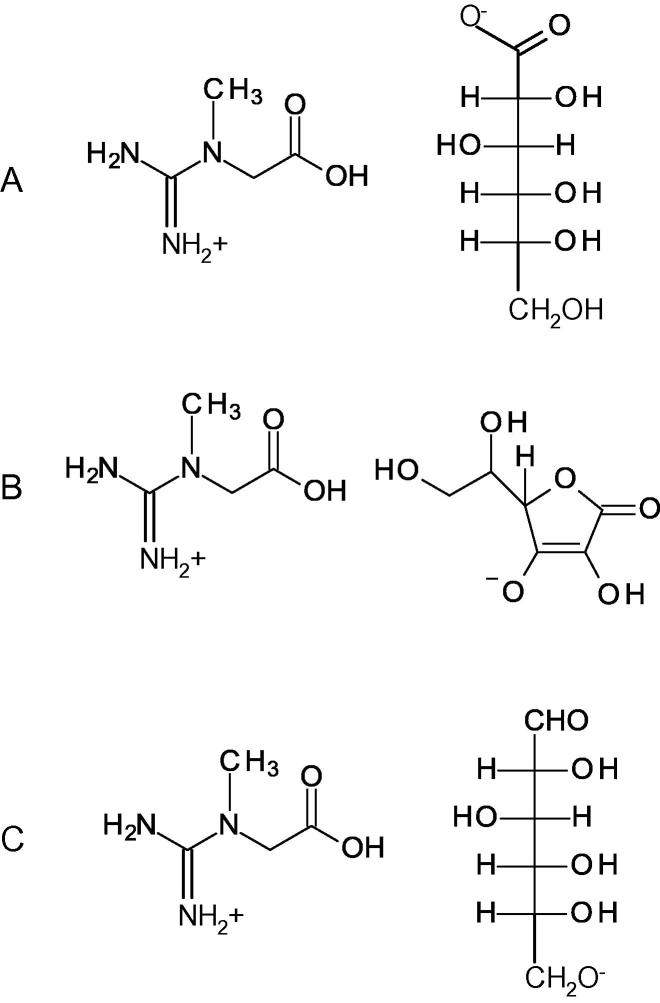
(a) Creatine gluconate, (b) Creatine ascorbate, (c) Creatine glucose.

Creatine gluconate (Fig. 1a) and creatine glucose (Fig. 1c) are two salts formed by creatine and d-gluconic acid and by creatine and glucose, respectively. Creatine ascorbate (Fig. 1b) is a salt formed by creatine and ascorbic acid (Vitamin C). The creatine-ascorbate and creatine glucose were synthesized following the method described in the Experimental procedure. Creatine gluconate is commercially available (see “Experimental procedure” section).

**Fig. 2:**
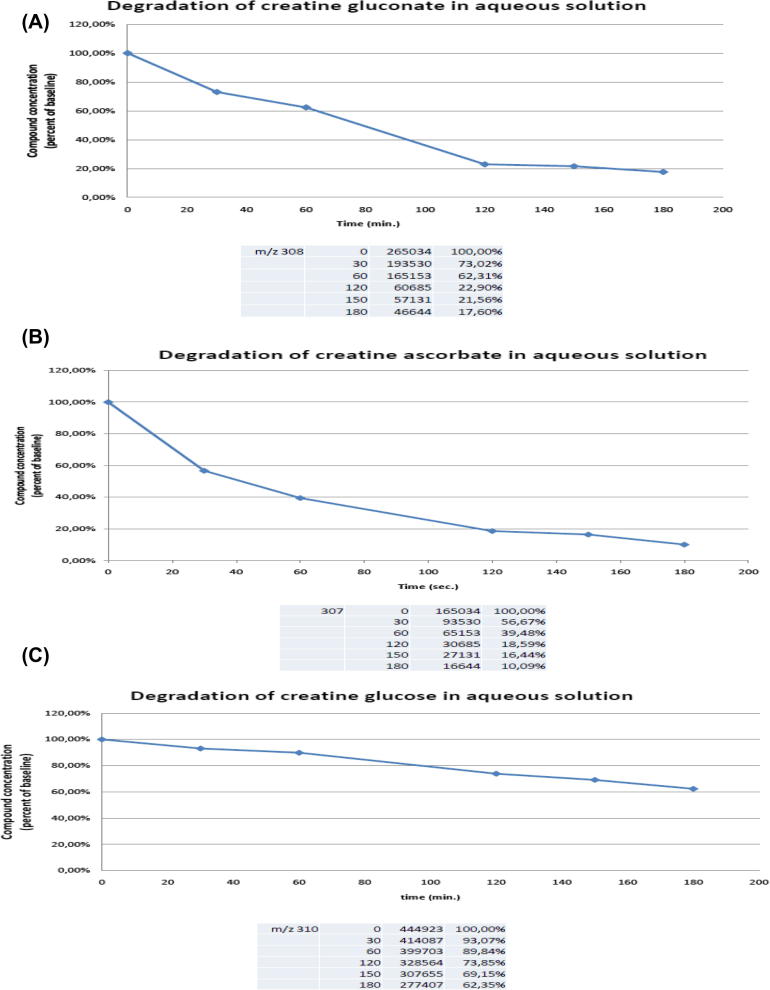
Compound degradation in the aqueous incubation medium. In ordinate substance concentration, normalized as described in the experimental procedures section. In abscissae time in minutes. Data are mean-relative standard deviation of three observations. (A) The original creatine glucose content is reduced to about half after about 75 min. After 3 h, only 20% of the initial compound amount is still present. (B) concentration of creatine ascorbate is reduced to about half after 40 min. After 3 h, only 5/7% of the initial compound amount is still present. (C) creatine glucose is the most stable of the three compounds, content is reduced to half after 180 min. After 3 h, 60% of the initial compound amount is still present.

We analyzed these creatine derivatives in HPLC-MS to certify the purity of the final products. We also investigated the stability of these salts in saline solution. We found that creatine glucose content is reduced to about half after about 75 min (Fig. 2A). Creatine ascorbate is reduced to about half after 40 min (Fig. 2B). Creatine glucose is the most stable of the three compounds, its content being reduced to half after 180 min. (Fig. 2C). Creatine monohydrate by contrast is rather stable in an aqueous solution, at least for a few hours (Adriano et al., 2011).

### Effects on tissue concentrations of creatine and of phosphocreatine

In these experiments, we tested creatine, creatine-gluconate, creatine-ascorbate and creatine-glucose under normal conditions and after block of the creatine transporter to assess the tissue creatine and phosphocreatine concentration.

#### Effects under normal conditions

**Fig. 3:**
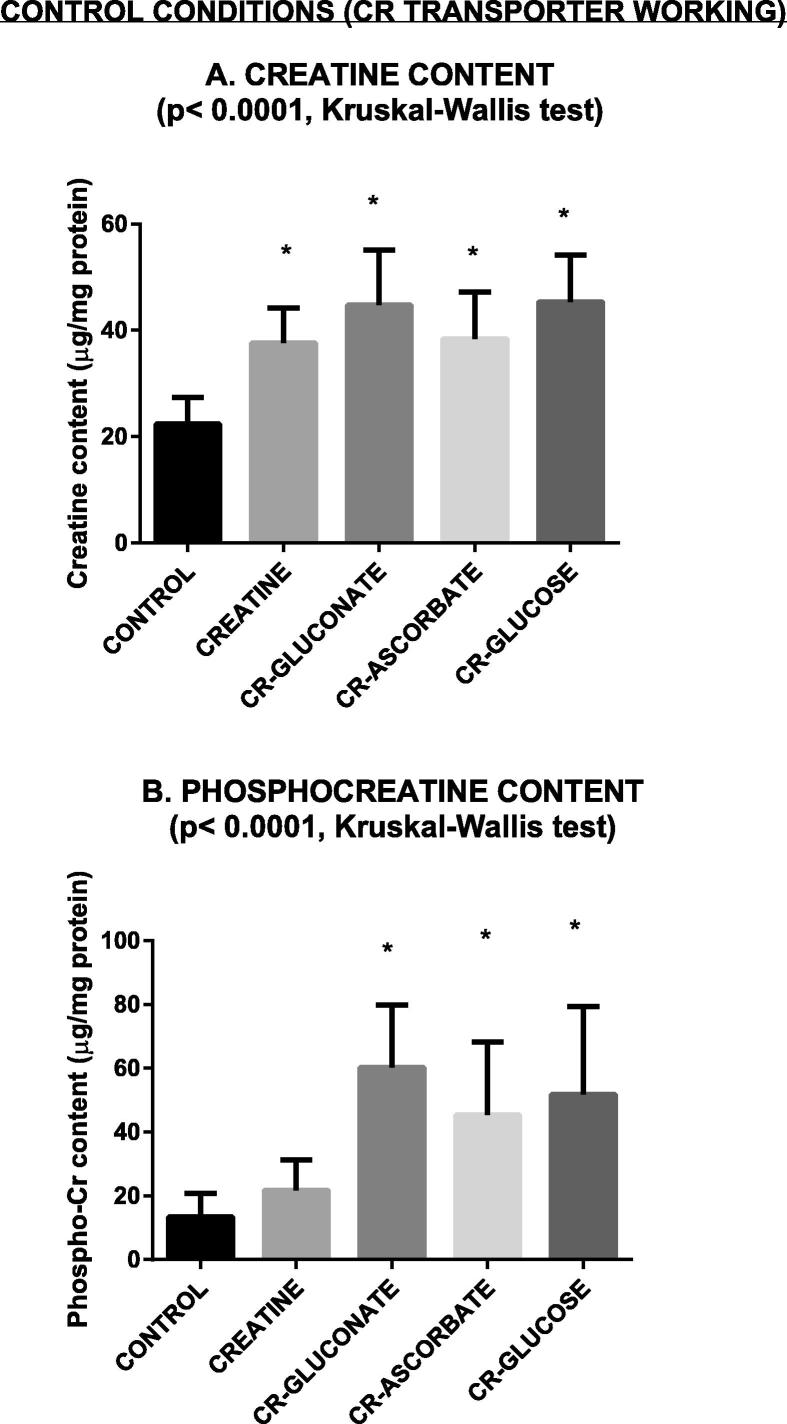
Creatine and phosphocreatine content under conditions of normally working transporter. CR = Creatine. Data are mean ± standard deviation. Asterisks show statistically significant differences with control group (*p* < 0.05, post hoc Dunn’s multiple comparison test). *N* = 10 in all groups.

Under control conditions, i.e. in slices where the creatine transporter is normally working, all compounds were able to increase tissue creatine concentration, with no differences among them (Fig. 3A). Moreover, all of them were also able to increase phosphocreatine concentration except creatine itself, which showed only a not significant trend toward increase (Fig. 3B).

#### Effects after block of the creatine transporter

**Fig. 4:**
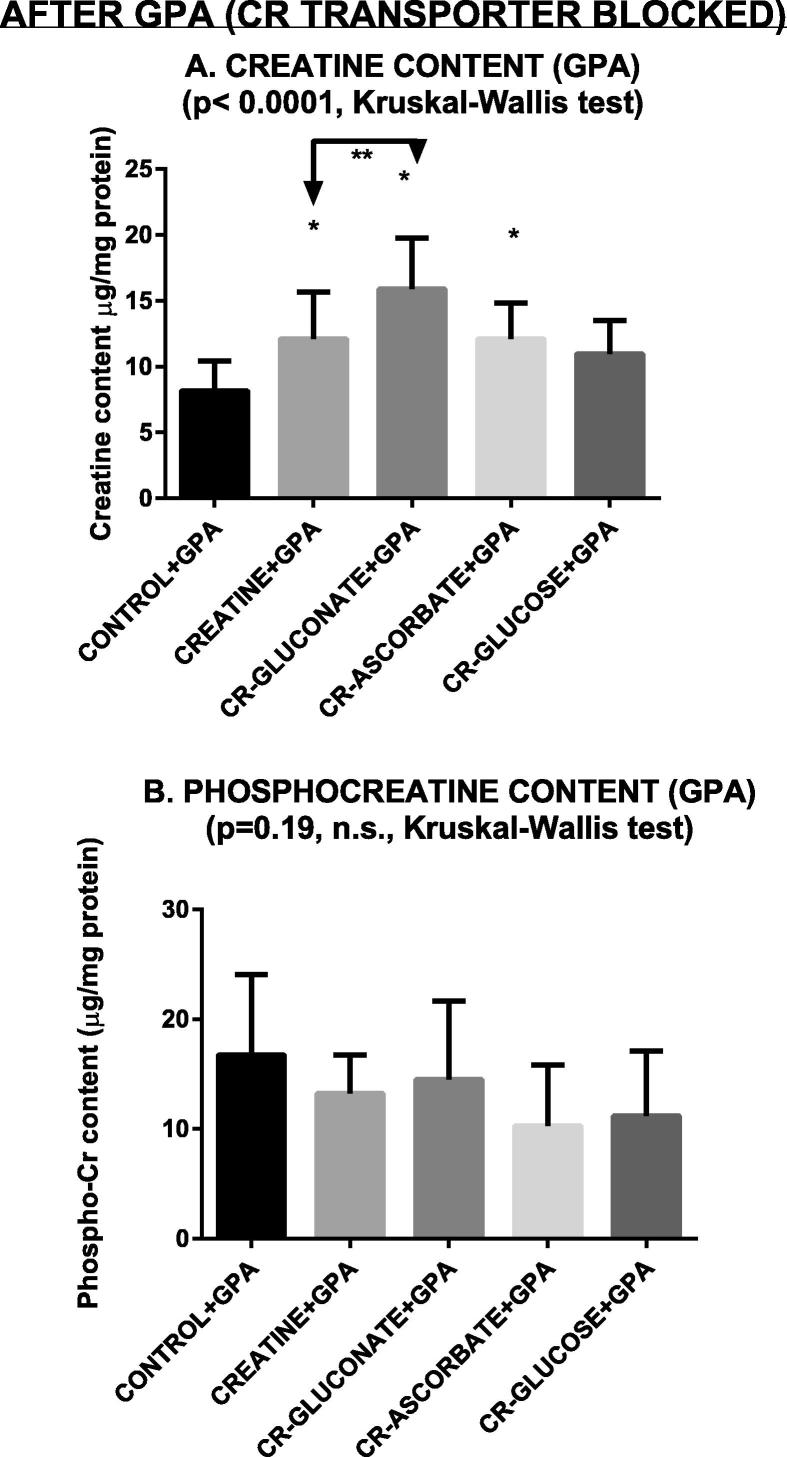
Creatine and phosphocreatine content under conditions of partially blocked transporter (GPA). CR = Creatine. Data are mean ± standard deviation. Asterisks show statistically significant differences with control group (*p* < 0.05, post hoc Dunn’s multiple comparison test). *N* = 10 in all groups.

After block of the creatine transporter with guanidine acetic acid (GPA) 10 mM, creatine, creatine-gluconate and creatine-ascorbate increased the tissue content of creatine, with creatine gluconate increasing tissue creatine more than creatine itself (Fig. 4A). Creatine-glucose was ineffective while only a slight increase was observed with creatine-ascorbate (Fig. 4A). No molecule was able to increase tissue content of phospho-creatine in these experimental conditions (Fig. 4B).

### Effects on disappearance of evoked potentials during anoxia

In these experiments, we tested creatine, creatine-gluconate and creatine-ascorbate under normal conditions and after block of the creatine transporter to evoke electrical potentials during anoxia. We were discouraged from testing creatine-glucose by the fact that the latter was ineffective in increasing creatine content after transporter impairment (see above).

#### Effects under normal conditions

**Fig. 5:**
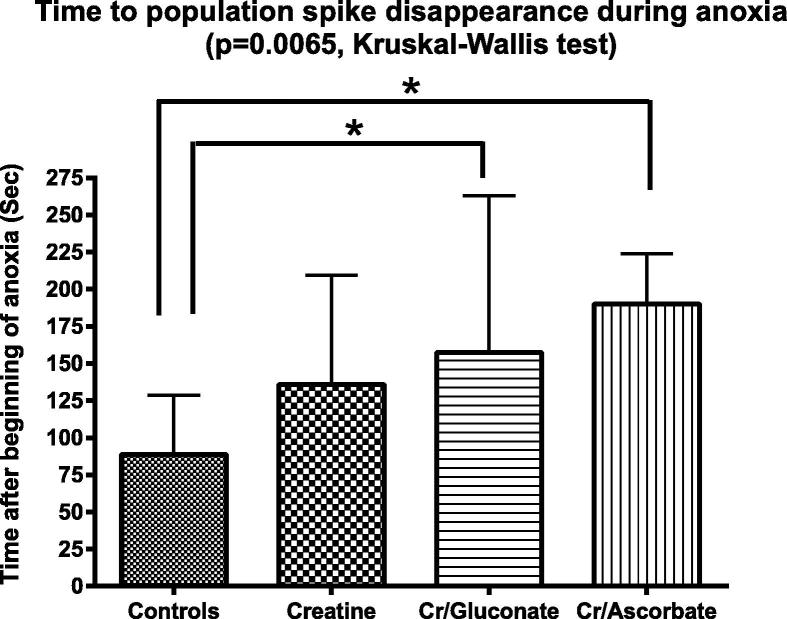
Time to disappearance of the population spike during anoxia (the latter was obtained by replacing oxygen with nitrogen in the incubation chamber). Abbreviations Cr = creatine; Cr/gluconate = creatine gluconate; Cr/ascorbate = creatine ascorbate. Data are mean ± standard deviation. Asterisks above brackets show statistically significant differences with control group (*p* < 0.05, post hoc Dunn’s multiple comparison test). *N* = 22–26 (min–max in all groups).

Under baseline conditions, both creatine ascorbate and creatine gluconate were able to delay population spike disappearance during anoxia (Fig. 5). Creatine did not provide a statistically significant effect, even if a not significant trend toward delay was observed (Fig. 5).

#### Effects after block of the creatine transporter

**Fig. 6:**
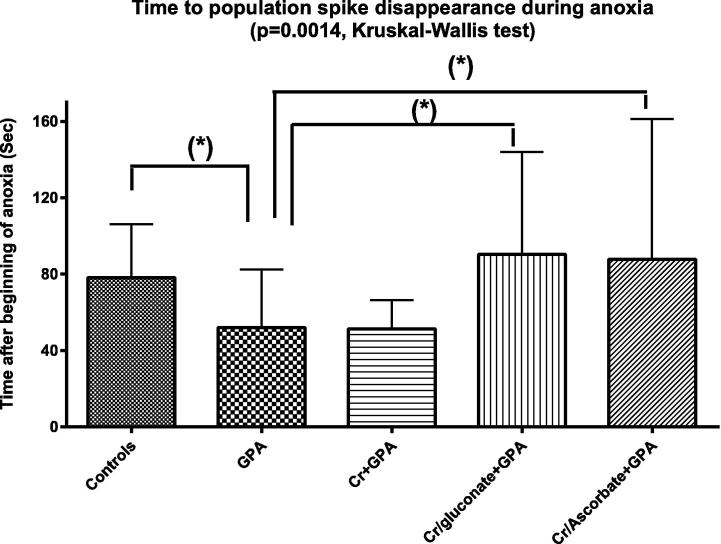
Time to disappearance of the population spike during anoxia (the latter was obtained by replacing oxygen with nitrogen in the incubation chamber). Abbreviations: GPA = guanidine-propionic acid (blocker of the creatine transporter), Cr = creatine; Cr/gluconate = creatine gluconate; Cr/ascorbate = creatine ascorbate. Data are mean ± standard deviation. Asterisks above brackets show statistically significant differences with the GPA group (*p* < 0.05, post hoc Dunn’s multiple comparison test). *N* = 14–17 (min–max in all groups).

After impairment of the creatine transporter with 1 mM GPA (Fig. 6), population spike disappeared faster than in normal slices (compare controls with GPA in Fig. 6). This suggests that the block of the creatine transporter by itself has a deleterious effect on anoxia survival. In the presence of GPA, creatine monohydrate was again unable to delay PS disappearance, while both creatine ascorbate and creatine gluconate did delay such disappearance (Fig. 6).

Table 1 summarizes the above-reported effects of the different creatine-derived molecules on the various parameters we investigated

## Discussion

Since creatine appears as an internal salt and is only a relatively weak base, it is rather unusual that stable creatine salts are formed with acid enols like ascorbic acid, and with mild organic acids like gluconic acid or glucose. In all these salts, the non-creatine compound is transported across biological membranes (BBB and the cells’ plasma membrane) by a specific transporter. We hypothesized that the creatine molecule in each of these salts may be transported across the same biological membranes together with the linked compound. Such a mechanism has been demonstrated before for other molecules (Greer M. Carrier drugs. Presidential address American Academy of Neurology, 1987. Neurology. 1988 Apr;38(4):628–32). Specifically, with this same mechanism ascorbic acid has been shown to be an efficient vector for many pharmaceuticals, transporting across the BBB antiepileptic, antiviral and antitumoral compounds (Dalpiaz et al., 2005). Such transport is mediated by SVCT2, the ascorbic acid transporter.

Thus, in principle one or more of the glucose transporters should carry the creatine glucose salt. There are six glucose transporters (GLUTS) but only four have been detected in high dose in brain: GLUTS 1, 3, 5 and 7 (Simpson et al., 1994). GLUT 2 appears to be widely expressed in all brain regions, but at apparently low levels, GLUT 4 is expressed in the pituitary, the hypothalamus and the medulla (Brant et al., 1993). GLUT1 have two isoform: molecular mass 55 kDa present in endothelial cells and 45 kDa present in the astroglia and neurons. the concentration of 55 kDa GLUT1 in the BBB, which has been found by CH binding to be of the order of 60–80 pmol/mg, with a dissociation constant (K,) for CH of 0.8 pM in purified human microvessels (Dick et al., 1984). GLUT3 is present in cell membranes that make up cell bodies, dendrites and axons of neurons its widespread distribution in neuronal elements including the neurohypophysis and cerebellar granule, and its absence in white matter, adenohypophysis, pineal gland and cerebral microvessels (Brant et al., 1993). GLUT5 to be present in resident microglial cells, in macrophages functionally and antigenically related to microglia. GLUT 7 has been detected in a certain sub-population of astrocytes (Simpson et al., 1994). Moreover, we hypothesize that d-gluconic acid may be transported by one or more of the same glucose transporters (a specific gluconate transporter has been so far found only in prokaryotes) due to their chemical similarity. By contrast, ascorbic acid has a specific transporter (Rivas et al., 2008) that should in theory carry the creatine ascorbate salt.

Under normal conditions (Cr transporter working) exogenous creatine increased creatine intracellular content as expected (Fig. 3A), although it did not increase phosphocreatine content (Fig. 3B). The latter finding may be due to the relatively low concentration of creatine we used. In fact, Bianchi et al. (2007) demonstrated that increase of phosphocreatine in the human brain *in vivo* is dependent on the dose of exogenous creatine (cf. for example their Fig. 3C). Thus, it is possible that the 2 mM creatine concentration we used was not sufficient to increase phosphocreatine in our slices. Whatever its explanation, lack of phosphocreatine increase in our experiments was very likely the reason why creatine in our hands did not delay disappearance of synaptic transmission during anoxia (Fig. 5), an effect that Whittingham and Lipton (1981) found after incubating brain slices with 25 mM creatine.

Under conditions of pharmacologically impaired transporter (Fig. 4) creatine again increased to an extent its own tissue content (Fig. 4A). This finding suggests that GPA caused only an incomplete block of the transport. This is consistent with what some of us previously published (Lunardi et al., 2006). In that reference some of us found that 10 mM GPA reduces to 50% (thus it does not fully prevent) creatine uptake by brain slices (cf. Fig. 4A of the above reference).

In the present experiments, creatine supplementation after albeit partial transporter block with GPA did not increase phosphocreatine concentration (Fig. 4B). Consequently, and as expected, creatine was not able to delay PS disappearance during anoxia after transporter impairment (Fig. 6).

Concerning creatine salts, under normal conditions (transporter working regularly) all of them increased creatine content as creatine did (Fig. 3A). However, at variance with creatine they also significantly increased tissue phosphocreatine content (Fig. 3B), perhaps suggesting a better penetration of these compounds across the cell plasma membrane. We also can hypothesize that the three compounds are easily phosphorylated (then probably degraded to phosphocreatine) but not so easily de-phosphorylated. This is in fact what happens with cyclocreatine, whose dephosphorylation is slower than phosphorylation (LoPresti and Cohn, 1989). This would not be surprising, as in normal conditions these compounds should enter cells using both creatine and glucose or ascorbate transporters. Alternatively (or additionally), we may speculate that the presence in the salt of the anionic portion (glucose, gluconic and ascorbic acid) can also create a protective shell to the creatine molecule. This protective shell could prevent or delay transformation of creatine into creatinine. Thus, creatine would be slowly released being available for a longer time to the subsequent phosphorylation.

Probably because of the increase in phosphocreatine they determined, creatine-gluconate and creatine-ascorbate also delayed the disappearance of evoked electrical potentials during anoxia (Fig. 5).

Under conditions of transporter impairment, creatine ascorbate and creatine gluconate increased again the intracellular content of creatine, while creatine-glucose did not have such effects (Fig. 4A). Moreover, creatine-gluconate increased tissue creatine significantly more than creatine itself did. The latter findings again strongly suggests that these creatine-derived compounds enter neurons independently on the Cr transporter. We hypothesize that creatine gluconate enters the cells using one or more of the glucose transporters, and that creatine-ascorbate enters the cells using the SVCT2 (ascorbic acid) transporter. After entry, they are likely split back to their two constitutive molecules (creatine and ascorbate, or creatine and gluconate).

Despite being salts, thus dimeric molecules whose two constituents are not covalently bound, these compounds decayed slowly is aqueous solution (Fig. 2). Such a slow decay is consistent with the biological effects we found.

We do not exclude a partial dissociation of the salts in two components into ACSF medium. However, this could possibly explain the electrophysiological effects (Fig. 2, Fig. 3) not the biochemical ones. In fact, these substances raise the levels of phosphocreatine significantly, something that creatine alone does not do (Fig. 4). Moreover, a specific compound (creatine gluconate) raises the creatine levels more than creatine itself when the transporter is blocked (Fig. 5). This effect can be explained only by a better penetration by the salts, not by a separate entry of the two moieties.

None of the tested salts should be toxic, since in them creatine is bound to compounds that are used in human diet. Indeed, creatine gluconate is currently sold as a dietary supplement for humans (Myprotein, Manchester UK). Thus, these creatine-derived compounds may be useful in the treatment of hereditary CrT transporter deficiency. A potential pitfall might be, however, their route of administration. In fact, being salts they are likely not absorbed as such by the intestinal mucosa. If this was the case, they might not reach as salts the BBB, or they might reach it in an amount insufficient to provide a biological effect. Further research is needed to investigate this issue. However, it is likely that a parenteral way of administration might be necessary. Alternatively, dimeric molecules where the two compounds are covalently bound might be effective even by oral route.

## Conclusions

After the creatine transporter was partially impaired by a submaximal dose of GPA, creatine gluconate is capable to increase creatine content more than creatine does, and to delay synaptic failure during anoxia (an effect that creatine does not have). Therefore, it might be useful in the therapy of creatine transporter deficiency, a currently incurable condition. The results we report justify further research on this topic.

## Experimental procedure

### Experimental compounds

Creatine monohydrate was purchased from Sigma–Aldrich (Hercules CA, USA). Creatine gluconate (Fig. 1) was purchased from Myprotein (Meridian House, Gadbrook Park, Gadbrook Way, Northwich, UK). HPLC check of purity yielded >99%.

Creatine-ascorbate and Creatine-glucose was synthesized following the method described by Pischel et al. (1999) with some modifications.

The partner compound (ascorbic acid or glucose) and creatine monohydrate in equivalent molar ratio were suspended in ethyl acetate protected from light. The reaction was carried out at room temperature until its completion monitoring by thin layer chromatography (TLC). Two eluent phases were used for the TLC: n-butanol:CH3COOH:H2O (6:2:2) and MeOH:CHCl3:CH3COOH (8:1.75:0.25). Detection was obtained with a solution of ninhydrin in ethanol spraying before putting on hot plate over 100 °C.

Then, at the completion of the reaction (about 24 h), the mixture was filtered and washed twice with ethyl acetate. The white powder obtained was dried in a vacuum chamber until dryness. The yield was almost quantitative.

The final product was finally verified by HPLC–MS analysis. The qualitative analysis of the products was finally confirmed by ESI-MS and ESI-MS^2^ analysis. Molecular weights were: *m*/*z* = 307.2 for creatine-ascorbate and *m*/*z* 311.2 for Creatine-glucose. The products were used in *in vitro* experiments without further purification.

### Stability in aqueous medium of creatine ascorbate, creatine gluconate and creatineglucose

To investigate the stability of the three creatine salts, creatine ascorbate, creatine gluconate and creatine glucose, the constructs were dissolved at the final concentration of 2 mM in ACSF at 36 °C and the solutions were diluted 500 times in waters. 10 microliters of each solution were injected in the MS system by flow injection analysis (FIA), and the obtained peaks were quantified. We were interested to know the amount of creatine ascorbate, creatine gluconate and creatine glucose that was still intact at time 0 and after 60, 120 and 180 min of treatment. Thus, at these times, the area under each peak was measured and normalized as a percentage of the peak area relative to each compound, at time 0 min.

### Preparation of hippocampal slices

Approval to conducting the experiments was obtained from the Italian Ministry of Health (Ministero della Salute). Experiments were carried out in compliance with animal care requirements requested by Italian law (law D.L. 27.1.1992 n. 116, in agreement with the European Union directive 86/609/CEE). Hippocampal slices were prepared *in vitro* as previously described (Adriano et al., 2011) In order to isolate the hippocampus, the animal is anesthetized with ethyl ether and immediately decapitated. After this the left hippocampus is extracted and subdivided into transversal slices with respect to the length of the organ, the slices being 600-μm thick for electrophysiological experiments or 400 μm for biochemical experiment. The slices thus obtained are transferred into a serum-free solution of artificial cerebrospinal fluid which is constantly oxygenated by a mixture of 95% oxygen and 5% carbon dioxide, in this way maintaining the pH around 7.35–7.4, and are incubated in a thermostatic bath at a temperature of 32 °C for electrophysiological experiments or 36 °C for biochemical experiment. The artificial cephalorachidian fluid (ACSF – Artificial Cerebro – Spinal Fluid) is an aqueous solution made up of the following compounds: NaCl 130 mM; KCl 3.5 mM; NaH_2_PO_4_ 1.25 mM; NaHCO 24 mM; CaCl 31.2 mM; MgSO 21.2 mM; Glucosio 410 mM (pH 7.35–7.4)

### Inactivation of creatine transporter in hippocampal slices

To functionally inactivate the creatine transporter we used guanidinopropionic acid (GPA) as described in our previous work (Lunardi et al., 2006). We could not use chloride-free incubation medium as a method to block creatine transporter nor we could use higher concentrations of GPA (1 Mm for electrophysiological experiments or 10 Mm for biochemical experiment) because under both treatments (chloride-free incubation or GPA>1 mM) no electrical activity could be elicited from the slices (preliminary results, not shown). Our previous data showed that incubation with GPA 1 mM reduced baseline creatine content by about 50% (Lunardi et al., 2006, Fig. 4) and reduced uptake of creatine by about the same measure (Lunardi et al., 2006, Fig. 5A).

### Study of the neuroprotective effect

The neuroprotective effect of the compounds deriving from creatine was studied using electrophysiological techniques on *in vitro* hippocampus slices. We followed a published method (Whittingham and Lipton, 1981) that we previously used in our laboratory (Balestrino et al., 1999, Perasso et al., 2008). It is based on Whittingham and Lipton’s observation, who showed that treatment prevented the irreversible loss of the synaptic transmission in mouse hippocampal slices during hypoxia, a protective effect that was attended by sparing of ATP during hypoxia itself (In the slices that underwent anoxia and treatment with creatine, the ATP concentration had lowered itself to 7.9 μmol/gr of proteins while in the control slices to 3.6 μmol/gr of proteins. The pre-anoxic ATP content was 13.9 μmol/gr of proteins).

To carry out this type of experiment we used 4- week-old ICR male mice (CD-1) from Harlan farm, as the experimental model. Hippocampal slices were prepared following the method shown above. After 3 h of pre-incubation, the slices were transferred into an incubation and electrophysiological registering chamber (Fine Science Tools, Vancouver, Canada). The tissue is maintained at 35 ± 1 °C, with a constant flow (2 ml/min) of ACSF having the same composition as that in the incubation medium. After this anoxia is induced by substituting the oxygen present in gaseous phase in the incubation chamber with nitrogen, after which the action potential in the extracellular compound is measured, stimulating every 5 s. Two tungsten microelectrodes are positioned on the hippocampal slice, where one (with a stimulating function) is positioned in the Schaffer Collaterals, and the other (with a registering function) is positioned in the CA1 cellular body layer. Once anoxia is established, the P.S. tends to reduce progressively till it disappears. The amount of time the signal (P.S.) takes to disappear from the moment anoxia starts is registered. The neuroprotective activity of creatine or of compounds with a similar action as creatine is carried out by delaying the depletion of ATP during anoxia and thus delaying the disappearance of the P.S., which is ATP dependant (Whittingham and Lipton, 1981).

We set up the following experimental groups:

• ACSF.
• ACSF + creatine (2 mM).
• ACSF + the compound to be tested (2 mM).
• ACSF + GPA (1 mM).
• ACSF + GPA (1 mM) + Creatine (2 mM).
• ACSF + GPA (1 mM) + the compound to be tested (2 mM).

### Tissue processing for biochemical experiment

Slices 400- μm-thick were incubated at 36 °C in one of the following media:

• ACSF.
• ACSF + creatine (2 mM).
• ACSF + the compound to be tested (2 mM).
• ACSF + GPA (10 mM).
• ACSF + GPA (10 mM) + Creatine (2 mM).
• ACSF + GPA (10 mM) + the compound to be tested (2 mM).

To reduce possible sample variability due to the slicing procedure, we pooled slices from different animals before incubation. To this aim, slices from each animal were distributed among all the incubation beakers, dropping two slices from that animal in each of the incubation beakers. Thus, each beaker contained 16 hippocampal slices from ten different animals. Each beaker was filled with one of the above described incubation media and was considered as one experimental subject. After 180 min of incubation at 36 °C, slices from each beaker were washed with saline solution. The incubation times we used are compatible with an uptake of the three compounds into the tissue. Whittingham and Lipton (1981) showed that in brain slices tissue content of phosphocreatine increases linearly over 3-h of incubation with extracellular creatine. Their data show that even after 1 or 2 h of incubation the tissue levels of phosphocreatine are elevated above baseline. Since our compounds have a half-life of 1–2 h, the incubation time we used is compatible with an increased uptake of our compounds. They were then instantly frozen on the wall of an Eppendorf vial maintained in liquid nitrogen, and immediately stored at −80 °C. Afterward, the slices from each beaker were homogenized together in a solution of perchloric acid 0.3 M, to inactivate creatine kinase. The homogenate obtained was adjusted to pH 7 with potassium carbonate 3 M. Samples were centrifuged. Proteins in the precipitate were evaluated by bicinchoninic acid assay (Smith et al., 1985) using BCA Protein Assay Kit (Sigma–Aldrich); all the measures were performed using bovine serum albumin (BSA) as standard. The protein concentration was used to normalize the levels of different metabolites in the supernatant.

### Tissue content of creatine and phosphocreatine

The quantitative analysis of metabolites was carried out, with minor modifications, according to a previous published paper (Lunardi et al., 2006), using high-performance liquid chromatography (Agilent 1260system – Agilent Technologies, Palo Alto, CA, USA) equipped with a standard autosampler. Briefly, separations were performed on a Symmetry C18, 3.9 mm x 150 mm column with 5-μm particle size (Waters Corporation, Milford, MA, USA) in isocratic mode. The mobile phase was an aqueous solution of 40 mM sodium phosphate dibasic (Na_2_HPO_4_) containing 1 mM tetrabutylammonium hydrogen sulfate (TBAHS) (solvent A) while the mobile phase B was an aqueous solution of sodium phosphate monobasic (NaH_2_PO_4_) 40 mM, TBAHS 1 mM, at pH 6.8. All reagents were of analytical grade (Sigma, St. Louis, MO, USA). Flow rate was 0.8 ml/min and wavelength was set to 220 nm. The column temperature was set to 30 °C. Injection volume was 5 μl both for standards and samples. Standard molecules were diluted in the mobile phase to reach, time by time, the desired concentration and supernatant of sample were diluted 1:1 in the mobile phase. The calibration curves, performed in triplicate using three standard concentrations including the expected values for samples, were carried out before each set of samples and accuracy was verified “in day”. Sample concentrations were determined using sample peak areas. The accuracy was in the range of ±13 to ±16% at the tested levels and the relative standard deviation was in the range of 8% to 12%.

## Figures and Tables

**Table 1:** Summary of the various effects by the compounds we investigated

Compound	Transporter working			Transporter impaired (GPA)		
	Increase in tissue Cr content	Increase in tissue PCr content	Delay in PS disappearance during anoxia	Increase in tissue Cr content	Increase in tissue PCr content	Delay in PS disappearance during anoxia
Creatine	Yes	No	no	Yes	No	No
Cr-gluconate	Yes	Yes	Yes	Yes (more than creatine itself)	No	Yes
Cr-ascorbate	Yes	YES	Yes	Yes	No	Yes
*Cr-glucose*	*Yes*	*Yes*	*N/A*	*No*	*No*	*N/A*
